# Naringenin Inhibits Superoxide Anion-Induced Inflammatory Pain: Role of Oxidative Stress, Cytokines, Nrf-2 and the NO−cGMP−PKG−K_ATP_Channel Signaling Pathway

**DOI:** 10.1371/journal.pone.0153015

**Published:** 2016-04-05

**Authors:** Marília F. Manchope, Cássia Calixto-Campos, Letícia Coelho-Silva, Ana C. Zarpelon, Felipe A. Pinho-Ribeiro, Sandra R. Georgetti, Marcela M. Baracat, Rúbia Casagrande, Waldiceu A. Verri

**Affiliations:** 1 Departamento de Ciências Patológicas, Centro de Ciências Biológicas, Universidade Estadual de Londrina, Londrina, Brazil; 2 Departamento de Ciências Farmacêuticas, Centro de Ciências de Saúde, Universidade Estadual de Londrina, Londrina, Brazil; French National Centre for Scientific Research, FRANCE

## Abstract

In the present study, the effect and mechanism of action of the flavonoid naringenin were evaluated in superoxide anion donor (KO_2_)-induced inflammatory pain in mice. Naringenin reduced KO_2_-induced overt-pain like behavior, mechanical hyperalgesia, and thermal hyperalgesia. The analgesic effect of naringenin depended on the activation of the NO−cGMP−PKG−ATP-sensitive potassium channel (K_ATP_) signaling pathway. Naringenin also reduced KO_2_-induced neutrophil recruitment (myeloperoxidase activity), tissue oxidative stress, and cytokine production. Furthermore, naringenin downregulated KO_2_-induced mRNA expression of gp91phox, cyclooxygenase (COX)-2, and preproendothelin-1. Besides, naringenin upregulated KO_2_-reduced nuclear factor (erythroid-derived 2)-like 2 (Nrf2) mRNA expression coupled with enhanced heme oxygenase (HO-1) mRNA expression. In conclusion, the present study demonstrates that the use of naringenin represents a potential therapeutic approach reducing superoxide anion-driven inflammatory pain. The antinociceptive, anti-inflammatory and antioxidant effects are mediated via activation of the NO−cGMP−PKG−K_ATP_ channel signaling involving the induction of Nrf2/HO-1 pathway.

## Introduction

Pain is an unpleasant sensory and emotional experience, generally in association with tissue injury. During inflammation, pro-inflammatory mediators activate resident cells, recruited cells and nociceptors, thereby driving pain signaling. Nociceptive neurons do not express receptors for all inflammatory molecules, suggesting both direct and indirect activation and sensitization of nociceptors [1]. Increased levels of oxidative stress during the inflammatory response also contribute to nociception. For instance, reactive oxygen species (ROS) and reactive nitrogen species (RNS) can act directly and indirectly to induce nociceptor sensitization and activation [2–5]. The superoxide anion (O_2_^−^) is a common form of ROS that can drive nociception [5,6]. O_2_^−^ reacts with nitric oxide (NO) generating peroxynitrite, which also contributes to nociception [3]. Superoxide dismutase (SOD), an enzymatic antioxidant, transforms superoxide anion in hydrogen peroxide, which may also induce nociception [2]. Therefore, O_2_^−^ is a crucial ROS to the biological underpinnings driving nociception. O_2_^−^ increases other pro-inflammatory effects, including increasing vascular permeability [7], inducing cytokine release [8,9] and increasing neutrophil recruitment [9,10], as well as provoking overt pain-like behavior and hyperalgesia [2–5]. In a physiological state, O_2_^−^ levels remain under control by the action of the endogenous antioxidant systems, including SOD, and the endogenous antioxidant reduced glutathione (GSH) [9]. However, the imbalance between oxidants and antioxidants during inflammation leads to oxidative stress. This is important, as inhibiting the production of pro-inflammatory cytokines and ROS limit the development of inflammatory pain [6,11–13].

Naringenin (4’,5,7-tryhidroxy-flavonone) is a flavonoid which belongs to flavonones class found in citric fruits, including lemon, orange, tangerine and grapefruit [14]. Naringenin inhibits the nociceptive responses in models of formalin-, acetic acid- and capsaicin-induced overt pain, as well as neuropathic pain [15–17]. Naringenin also inhibits inflammation by targeting cyclooxygenase (COX)-2 in ethanol-induced liver injury [18] and *in vitro* [19]. Moreover, naringenin inhibits phosphorylation of nuclear factor kappaB (NFκB) subunit p65 and mitogen-activated protein kinases (MAPK) in daunorubicin-induced nephrotoxicity [20] as well as inhibiting the EGFR-PI3K-Akt/ERK MAPK signaling pathway in human airway epithelial cells [21]. Naringenin also inhibits a number of aspects of oxidative stress, including lipid peroxidation and O_2_^−^ production, as well as restoring GSH levels in UVB-induced oxidative stress in the skin of Hairless mice [22]. Furthermore, naringenin increases SOD in an experimental stroke model, highlighting its wide-acting induction of endogenous antioxidants [23]. In agreement with such antioxidant effects, naringenin also induces nuclear factor (erythroid-derived 2)-like 2 (Nrf2)/ heme oxygenase (HO)-1 in CCl_4_-induced hepatic inflammation [24]. Some flavonoids can induce antinociception by activating the NO−cGMP−PKG−ATP-sensitive potassium channel (K_ATP_) signaling pathway [25–28]. Activating this signaling pathway is an important mechanism of action of a number of clinical analgesics, such as opioids [25], and some non-steroidal anti-inflammatory drugs including dipyrone [26], diclofenac [27], and indomethacin [28].

Given the above, the current study addresses the analgesic effects of naringenin in a model of O_2_^−^-triggered inflammatory pain. It was also investigated as to whether naringenin's mechanism of action involves the NO−cGMP−PKG−K_ATP_ channel signaling pathway, the regulation of inflammatory mediators/enzymes and oxidative stress as well as the transcription factor Nrf2, and its downstream target, HO-1.

## Materials and Methods

### Animals

Male Swiss mice (25 ± 5 g) from Londrina State University were housed in standard plastic cages with free access to food and water, with a light/dark cycle of 12:12 h, at 21°C. All behavioral testing was performed between 9 a.m. and 5 p.m. in a temperature-controlled room. At the end of experiments, mice were anesthetized with isoflurane 3% to minimize suffering (Abbott Park, IL, USA) and killed by cervical dislocation followed by decapitation. The animal condition was monitored daily and at indicated time points during the experiments. No unexpected animal deaths occurred during this study. Animals' care and handling procedures were in accordance with the International Association for Study of Pain (IASP) guidelines and with the approval of the Ethics Committee of the Londrina State University (process 133666.2013.71).

### Drugs and reagents

Potassium superoxide (KO_2_) (Alfa Aesar, 96,5%, Ward Hill, MA, USA). Naringenin (Santa Cruz Biotechnology, Inc., 98%, Dalla, TX, USA). Saline (NaCl 0,9%; Fresenius Kabi Brazil Ltda. Aquiraz, CE, Brazil). L-NAME (Research Biochemicals, Natick, MA, USA), KT5823 (Calbiochem, San Diego, CA, USA), ODQ [1H-(1,2,4)-oxadiazolol-(4,3-a)-quinoxalin-1-one, Tocris Cookson, Baldwin, MO, USA]. Glybenclamide, HTAB (Bromide, hexadecyl trimethyl ammonium), dihydrochloride O-dianisidine, GSH (reduced glutathione), EDTA sodium salt, ferric chloride hexahydrate, TPTZ (2,4,6-tripiridil-s-triazine) and Trolox (6-hydroxy-2,5,7,8-tetramethylchroman-2-11 carboxylic) were purchased from Sigma Chemical Co. (St. Louis, MO, USA). DMSO 2% and Tween 80 (Química Moderna, Barueri, SP, Brazil).

### Experimental procedures

Mice were pretreated per orally (po) with 16.7, 50, or 150 mg/kg of naringenin or with vehicle (saline) 1 h before intraplantar (ipl) or intraperitoneal (ip) injection of 30 μg or 1 mg of KO_2_, respectively [6]. The writhing response was evaluated between 0–20 min after KO_2_ ip injection and the paw flinching and licking nociceptive responses were quantified during 30 min after ipl injection of KO_2_. Mechanical and thermal hyperalgesia were evaluated 0.5–7 h after KO_2_. Inflammatory stimulation with KO_2_ induced mechanical and thermal hyperalgesia only in the ipsilateral paw, in the side where the stimulus was injected. Myeloperoxidase (MPO) activity was evaluated 7 h after KO_2_ administration. Mice were pretreated with inhibitors of the NO synthase (L-NAME; 90 mg/kg, ip, 1 h pre-treatment), guanylate cyclase (ODQ, 0,3 mg/kg, ip, 30 min pre-treatment), PKG (KT5823, 0,5 μg/animal, ip, 5 min pre-treatment), K_ATP_ channels (glibenclamide, 0,3 mg/kg, po, 45 min pre-treatment) before naringenin treatment (50 mg/kg, po). After 1 h mice received ipl injection of 30 μg of KO_2_, and mechanical and thermal hyperalgesia were assessed 0.5–7 h thereafter. Evaluations of oxidative stress (GSH, FRAP, TBARS, NBT) and cytokine production (tumor necrosis factor [TNF]α and interleukin [IL]-10) were carried out, as well as RT-qPCR measures of gp91^phox^, COX-2, prepro endothelin (ET)-1, Nrf2, HO-1, and IL-33, 3 h after KO_2_ injection in samples of paw skin.

### Overt pain-like behavioral tests

Abdominal writhing was induced by ip injection of 1 mg of KO_2_ [6]. Immediately after stimulus injection, each mouse was placed individually in a large glass cylinder, and the intensity of nociceptive behavior was quantified by counting the total number of writhings occurring between 0 and 20 min after stimulus injection. The writhing response consists of a contraction of the abdominal muscle together with a stretching of hind limbs, and the intensity was expressed as the cumulative number of abdominal contortions over 20 min. The number of paw flinches and the time spent licking the paw were determined between 0–30 min after ipl injection of 30 μg of KO_2_. Each mouse was placed in a large glass cylinder immediately after stimulus injection. The intensity of nociceptive behavior was quantified by counting the total number of paw flinches and the time (seconds) spent licking the ipsilateral paw [6].

### Mechanical hyperalgesia test

Mechanical hyperalgesia was evaluated by the electronic version of von Frey’s test, as reported previously [29]. In a quiet, temperature controlled room, mice were placed in acrylic cages (12 x 10 x 17 cm) with wire grid floors, 15–30 min before the start of testing. The test consisted of evoking a hind paw flexion reflex with a handheld force transducer (electronic anesthesiometer, IITC Life Science, Woodland Hills, CA) adapted with a 0.5 mm^2^ polypropylene tip. The investigator was trained to apply the tip perpendicularly to the central area of the plantar hind paw with a gradual increase in pressure. The gradual increase in pressure was manually performed in blinded experiments. The upper limit pressure was 15 g. The end-point was characterized by the removal of the paw followed by clear flinching movements. After paw withdrawal, the intensity of the pressure was automatically recorded, and the final value for the response was obtained by averaging three measurements. The animals were tested before and after treatments. The results are expressed by delta (Δ) withdrawal threshold (in grams) calculated by subtracting the mean measurements 0.5, 1, 3, 5 and 7 h after stimulus from the zero-time mean measurements [6].

### Thermal hyperalgesia test

Heat thermal hyperalgesia was performed using a hot plate at 55 ± 1°C [6]. The end-point was characterized by the removal of the paw followed by clear paw flinching or licking movements. The upper time was 15 s to avoid possible injury. The results are expressed by total withdrawal latency (in seconds) of measurements obtained 0.5, 1, 3, 5 and 7 h after stimulus [30].

### MPO assay

Neutrophil migration to the hind paw skin tissue was evaluated using an MPO kinetic-colorimetric assay, as described previously [6]. Samples of paw skin tissue were collected 7 h after stimulus in ice-cold 50 mM K_2_HPO_4_ buffer (pH 6.0) containing 0.5% hexadecyltrimethylammonium bromide (HTAB) and kept at -80°C until use. Samples were homogenized, centrifuged (16,100g x 2 min, 4°C), and the resulting supernatant was assayed for MPO activity spectrophotometrically at 450 nm (Multiskan GO Microplate Spectrophotometer, Thermo Scientific, Vantaa, Finland), with three readings in 1 min. The MPO activity of samples was compared to a standard curve of neutrophils. Briefly, 15 μL of sample was mixed with 200 μL of 50 mM phosphate buffer, pH 6.0, containing 0.167 mg/mL o-dianisidine dihydrochloride and 0.015% hydrogen peroxide. The results are presented as MPO activity (number of neutrophils X 10^10^ per g of tissue).

### GSH measurement

Paw skin sample was collected and maintained at -80°C for at least 48 h. Sample was homogenized with 200 μL of 0.02 M EDTA. The homogenate was mixed with 25 μL of 50% trichloroacetic acid and was homogenized three times during 15 min. The mixture was centrifuged (15 min x 1500 *g* x 4°C). The supernatant was added to 200 μL of 0,2 M TRIS buffer, pH 8.2, and 10 μL of 0,01M DTNB. After 5 min, the absorbance was measured at 412 nm against a reagent blank with no supernatant. A standard curve was performed with standard GSH. The results are expressed as GSH nmol per mg paw [31].

### FRAP assay

Paw skin sample was collected and immediately homogenized with 500 μL of 1.15% KCl, and was centrifuged (10 min x 200 *g* x 4°C). The ability of the sample to resist oxidative damage was determined using FRAP assays [32]. For the FRAP assay, 50 μL of supernatant was mixed with 150 μL of deionized water and 1.5 mL of freshly prepared FRAP reagent. The reaction mixture was incubated at 37°C for 30 min and the absorbance was measured at 595 nm. The results were equated against a Trolox standard curve (1.5–30 μmol/L, final concentrations). The results are expressed as Trolox equivalents per mg of paw.

### Superoxide anion production

The quantitation of O_2_^−^ production in tissue homogenates was performed using the NBT assay, as described previously [33]. Skin samples were collected 3h after the stimulus. Briefly, 50 μL of the homogenate was incubated with 100 μL of NBT (1 mg/mL) in 96-well plates at 37°C for 1h. The supernatant was carefully removed and the reduced formazan solubilized by adding 120 μL of 2M KOH and 140 μL of DMSO. The NBT reduction was measured at 600 nm using a microplate spectrophotometer reader (Multiskan GO, Thermo Scientific). The tissue weight was used for data normalization; thus the results are expressed as NBT reduction (OD/mg of paw).

### Lipid Peroxidation

Tissue lipid peroxidation was assessed by the levels of thiobarbituric acid reactive substances (TBARS) [34]. For this assay, TCA 10% was added to the homogenate and the mixture was centrifuged (1000 *g*, 3 min, 4°C) to precipitate proteins. The protein-free supernatant was then separated and mixed with TBA (0.67%). The mixture was kept in water bath (15 min, 100°C). Malondialdehyde (MDA), an intermediate product of lipid peroxidation, was determined by difference between absorbances at 535 and 572 nm using a microplate spectrophotometer reader. The results were presented as nmol of MDA per mg of paw [34].

### Cytokine measurement

Paw skin samples were collected 3 h after the injection of KO_2_, homogenized in 500 μL of ice-cold buffer containing protease inhibitors, centrifuged (3000 rpm x 10 min x 4°C) and the supernatants used to measure, TNFα and IL-10 levels, by an enzyme-linked immunosorbent assay (ELISA) using eBioscience kits. As a control, the concentrations of these cytokines were determined in animals injected with saline. The results are expressed as picograms (pg) of cytokine per mg of paw.

### Reverse transcription and quantitative polymerase chain reaction (RT- qPCR)

RT-qPCR was performed as previously described [33]. Paw skin samples were collected 3 h after stimulus and homogenized in trizol reagent, with the total RNA being isolated according to manufacturer’s directions. The purity of total RNA was measured with a spectrophotometer with the wavelength absorption ratio (260/280 nm) being between 1.8 and 2.0 for all preparations. Reverse transcription of total RNA to cDNA, and qPCR were carried out using GoTaq^®^ 2-Step RT-qPCR System (Promega) following the manufacturer’s instructions. The relative gene expression was measured using the comparative 2^−(ΔΔCq)^ method. The primers used were gp^91phox^, sense: 5’-AGCTATGAGGTGGTGATGTTAGTGG-3’, antisense: 5’-CACAATATTTGTAC CAGACAGACTTGAG-3’; IL-33, sense: 5’-TCCTTGCTTGGCAGTATCCA-3’, antisense: 5’-TGCTCAATGTGTCAACAGACG-3’; COX-2, sense: 5’-GTGGAAAAACCTCGTCCAGA-3’, antisense: 5’-GCTCGGCTTC CAGTATTGAG-3’; preproET-1, sense: 5’-TGTGTCTACTTCTGCCACCT-3’, antisense: 5’-CACCAGCTGCTGATAGATAC-3’; Nrf2, sense: 5’-TCACACGAGATGAGCTTAGGGCAA-3’, antisense: 5’-TACAGTTCTGGG CGGCGACTTTAT-3’; HO-1, sense: 5’-CCCAAAACTGGCCTGTAAAA-3’, antisense: 5’-CGTGGTCAGTCAACATGGAT-3’; β-actin, sense: 5’-AGCTGCGTTT TACACCCTTT-3’, antisense: 5’- AAGCCATGCCAATGTTGTCT-3’. The expression of β-actin mRNA was used as a reference gene to normalize data.

### Statistical analysis

Results are presented as means ± SEM of measurements made on six mice in each group per experiment, and are representative of two independent experiments. Two-way analysis of variance (ANOVA) was used to compare the groups and doses at all times (curves), when the hyperalgesic responses were measured at different times after the administration or enforcement of the stimuli. The factors analyzed were treatment, time, and time versus treatment interaction. When there was a significant time versus treatment interaction, one-way ANOVA followed by Tukey’s post hoc was performed on each occasion. On the other hand, when the hyperalgesic responses were measured once after the administration or enforcement of the stimuli, the difference between responses were evaluated by one-way ANOVA followed by Tukey’s post hoc. Statistical differences were considered to be significant at *p* <0.05.

## Results

### Naringenin inhibits KO_2_-induced overt pain-like behavior

**Fig 1:**
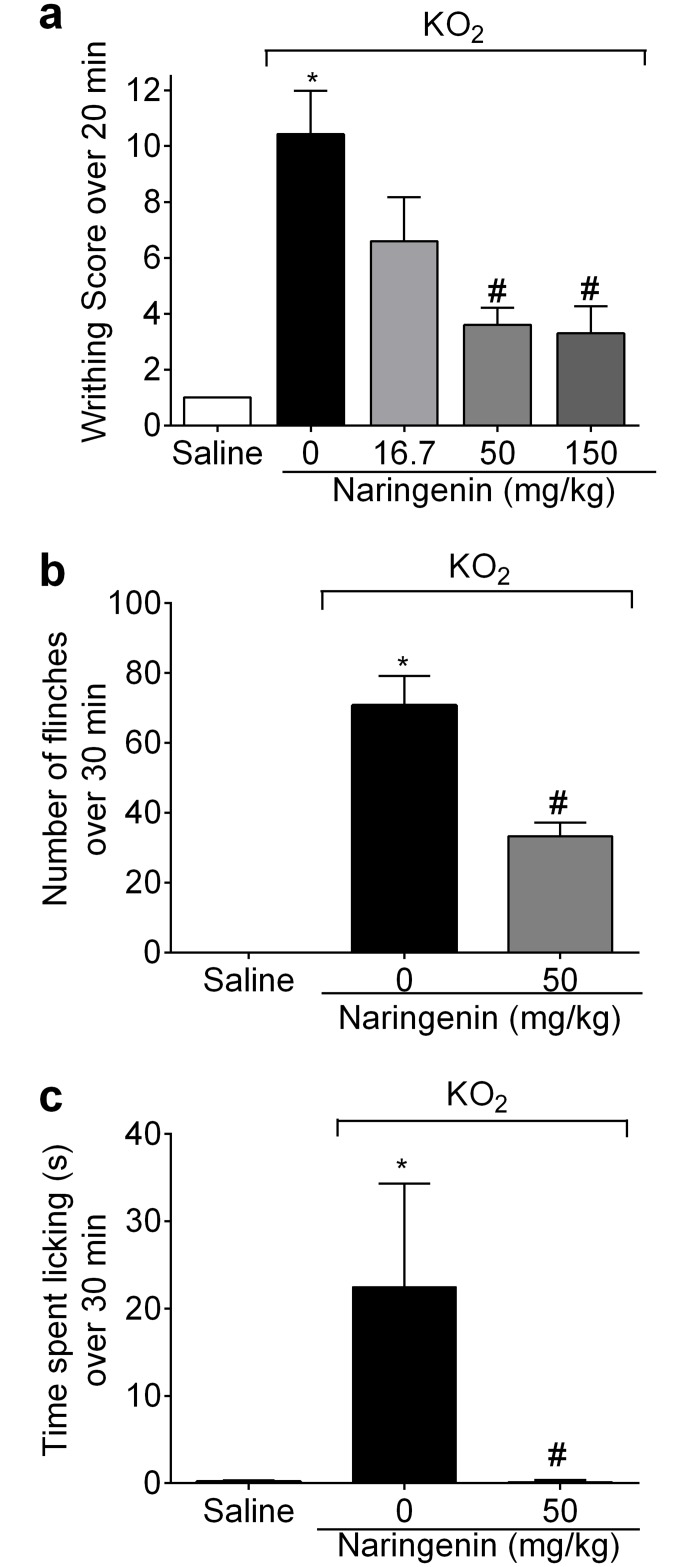
Naringenin inhibits KO_2_-induced overt pain-like behavior. (a-c) Mice received naringenin (16.7, 50 and 150 mg/kg, po) treatment 1 h before ip injection of 1 mg of KO_2_ or ipl injection of 30 μg of KO_2_. The total number of writhings was evaluated 0–20 min after ip injection of KO_2_. (b) The number of paw flinches and (c) time spent licking the paw were evaluated 0–30 min after ipl injection of KO_2_. Results are mean ± SEM of 6 mice per group per experiment, and are representative of 2 independent experiments. *p< 0.05 vs. saline group, #p< 0.05 vs. KO_2_ group. One-way ANOVA followed Tukey’s post hoc.

Mice received naringenin (16.7, 50,150 mg/kg, po) treatment 1h before injection of 1 mg of KO_2_ ip for the assessment of the total number of writhings, or received 30 μg of KO_2_ ipl for the evaluation of the total number of paw flinches and time spent licking the paw. The naringenin doses of 50 and 150 mg/kg inhibited KO_2_-induced writhing response at a similar magnitude of effect, with the 3 mg/kg dose showing no statistically significant effect (Fig 1a). The dose of 50 mg/kg of naringenin was therefore selected for the next experiments. Naringenin also inhibited KO_2_-induced paw flinches (Fig 1b) and time spent licking the paw (Fig 1c).

### Naringenin inhibits KO_2_-induced mechanical hyperalgesia, thermal hyperalgesia, and MPO activity

**Fig 2:**
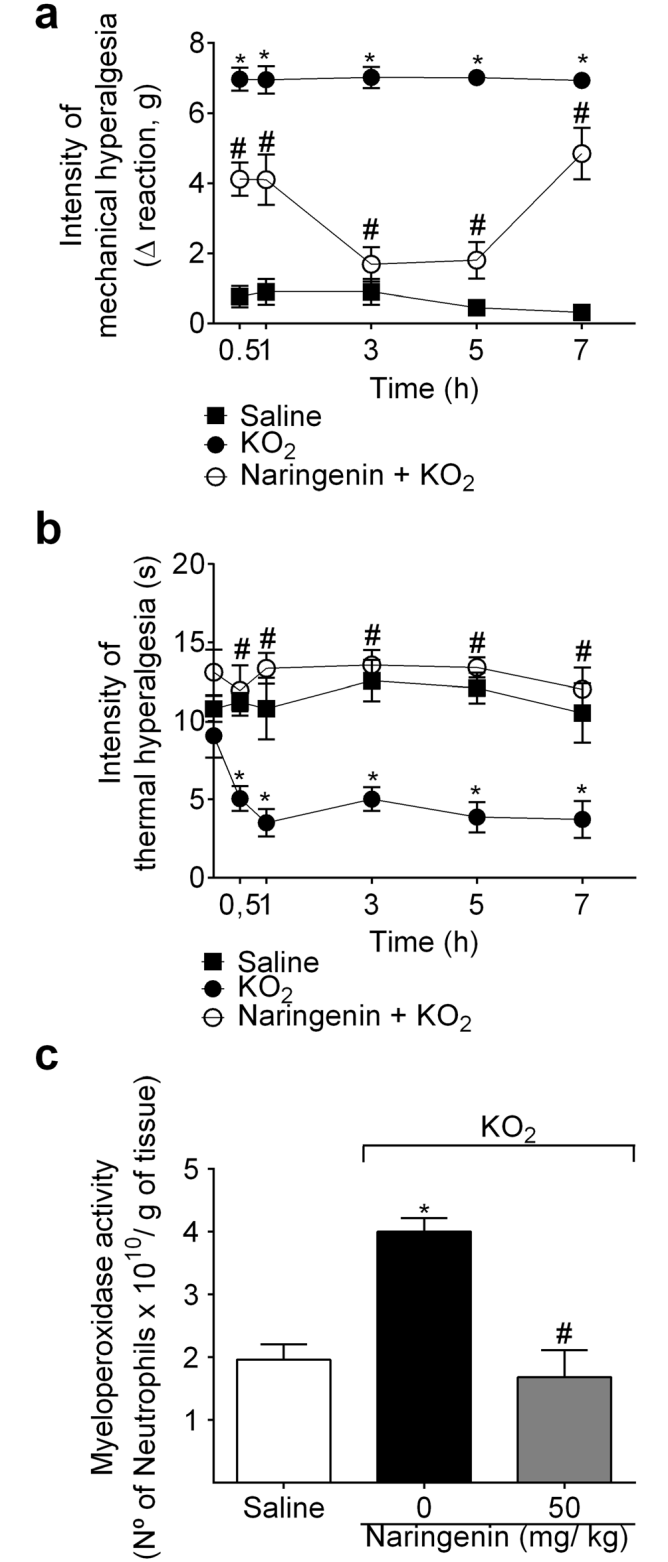
Naringenin inhibits KO_2_-induced mechanical hyperalgesia, thermal hyperalgesia and myeloperoxidase (MPO) activity. (a-c) Mice received naringenin (50 mg/kg, po) treatment 1 h before ipl injection of 30 μg of KO_2_. Mechanical (a) and thermal (b) hyperalgesia were evaluated between 0.5–7 h after ipl injection of KO_2_. (c) At the 7^th^ h after KO_2_ injection, paw skin samples were collected for MPO activity assay. Results are mean ± SEM of 6 mice per group per experiment, and are representative of 2 independent experiments. *p< 0.05 vs. saline group, #*p*< 0.05 vs. KO_2_ group. Repeated measures two-way ANOVA for hyperalgesia data and One-way ANOVA for MPO activity followed Tukey’s post hoc.

Mice received naringenin (50 mg/kg, po) treatment 1h before KO_2_ injection (30 μg, ipl). Mechanical and thermal hyperalgesia were assessed 0.5, 1, 3, 5 and 7 h after KO_2_ injection. Naringenin inhibited KO_2_-induced mechanical and thermal hyperalgesia at all time points (Fig 2a and 2b). Naringenin also reduced KO_2_-induced increased of MPO activity at 7 h (Fig 2c).

### Naringenin inhibits KO_2_-induced mechanical and thermal hyperalgesia by activating the NO−cGMP−PKG− K_ATP_ channel signaling pathway

**Fig 3:**
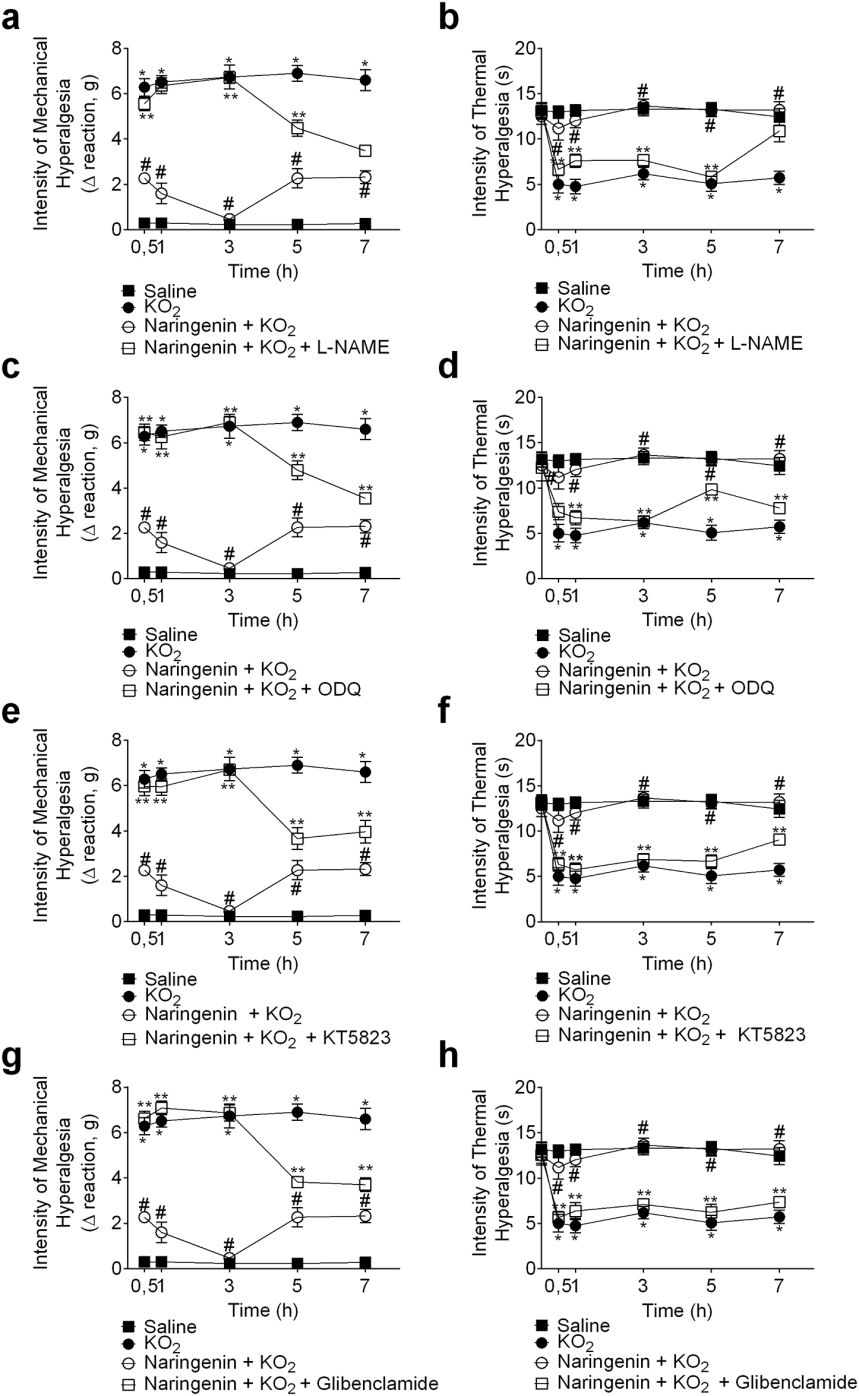
Naringenin inhibits KO_2_-induced mechanical and thermal hyperalgesia by activating NO/cGMP/PKG/K_ATP_ channel signaling pathway. Mice received (a,b) L-NAME (NOS inhibitor, 90 mg/kg, ip, 1 h), (c,d) ODQ (guanylate cyclase inhibitor, 0,3 mg/kg, ip, 30 min), (e,f) KT5823 (PKG inhibitor, 0,5 μg/mice, ip, 5 min), or (g,h) glibenclamide (K_ATP_ channel inhibitor, 0,3 mg/kg, po, 45 min) treatment before administration of naringenin (50 mg/kg, po). (a-h) 1 h after naringenin administration, mice received an ipl injection of 30 μg of KO_2_. Mechanical (a,c,e,g) and thermal (b,d,f,h) hyperalgesia were evaluated between 0.5–7 h after the ipl injection of KO_2_. Results are mean ± SEM of 6 mice per group per experiment, and are representative of 2 independent experiments. *p< 0.05 vs. saline group, #*p*< 0.05 vs. KO_2_ group. Repeated measures two-way ANOVA followed Tukey’s post hoc.

Mice were treated with inhibitors of a) NO synthase (L-NAME; 90 mg/kg, ip, 1 h pre-treatment), b) guanylate cyclase (ODQ, 0,3 mg/kg, ip, 30 min pre-treatment), c) PKG (KT5823, 0,5 μg/animal, ip, 5 min pre-treatment), and d) the K_ATP_ channel (glibenclamide, 0,3 mg/kg, po, 45 min pre-treatment) before naringenin (50 mg/kg, po, 1 h before KO_2_ injection) administration. After 1h naringenin treatment, mice received a KO_2_ ipl injection. L-NAME (Fig 3a and 3b), ODQ (Fig 3c and 3d), KT5823 (Fig 3e and 3f), and glibenclamide (Fig 3g and 3h) inhibited the analgesic effect of naringenin in KO_2_-induced mechanical and thermal hyperalgesia. Therefore, the anti-hyperalgesic mechanism of naringenin depends, at least in part, on activating the NO−cGMP−PKG− K_ATP_ channel signaling pathway.

### Naringenin inhibits KO_2_-induced oxidative stress

**Fig 4:**
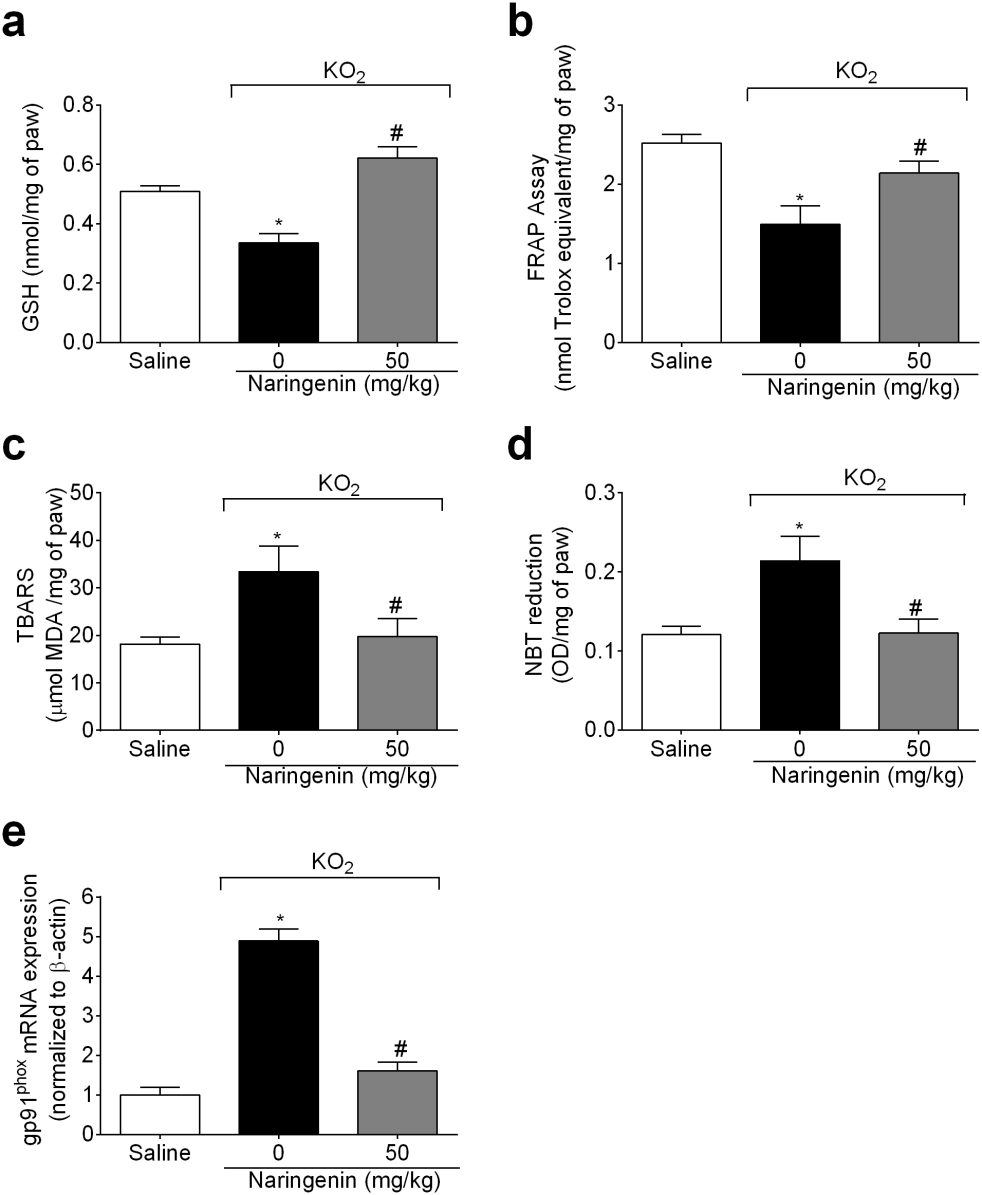
Naringenin inhibits KO_2_-induced oxidative stress and gp91^phox^ mRNA expression. (a-e) Mice received naringenin (50 mg/kg, po) treatment 1 h before ipl injection of 30 μg of KO_2._ Paw skin samples were collected 3 h after ipl KO_2_ injection. Sample analyses were (a) GSH levels, (b) total antioxidant capacity (FRAP assay), (c) lipid peroxidation (TBARS assay), (d) O_2_^−^ production (NBT assay), and (e) gp91^phox^ mRNA expression by RT-qPCR. β-actin was a reference gene to normalize mRNA expression data. Results are mean ± SEM of 6 mice per group per experiment, and are representative of 2 independent experiments. *p< 0.05 vs. saline group, #*p*< 0.05 vs. KO_2_ group. One-way ANOVA followed Tukey’s post hoc.

Mice received naringenin (50 mg/kg, po) treatment 1h before KO_2_ injection (30 μg, ipl). Samples of paw skin were collected after 3 h for colorimetric assays and RT-qPCR. KO_2_ depleted paw skin GSH (Fig 4a) and antioxidant capacity (Fig 4b); and increased lipid peroxidation (Fig 4c) and superoxide anion production (Fig 4d) in the paw skin. Naringenin treatment inhibited these deleterious effects of KO_2_ by restoring GSH levels (Fig 4a), and antioxidant capacity (Fig 4b), and inhibiting lipid peroxidation (Fig 4c) and O_2_^−^ production (Fig 4d). Corroborating the results on oxidative stress (Fig 4a–4d), naringenin also inhibited KO_2_-induced gp91^phox^ mRNA expression, a component of NADPH oxidase, an important source of O_2_^−^ production (Fig 4e).

### Naringenin inhibits KO_2_-induced cytokine production and mRNA expression

**Fig 5:**
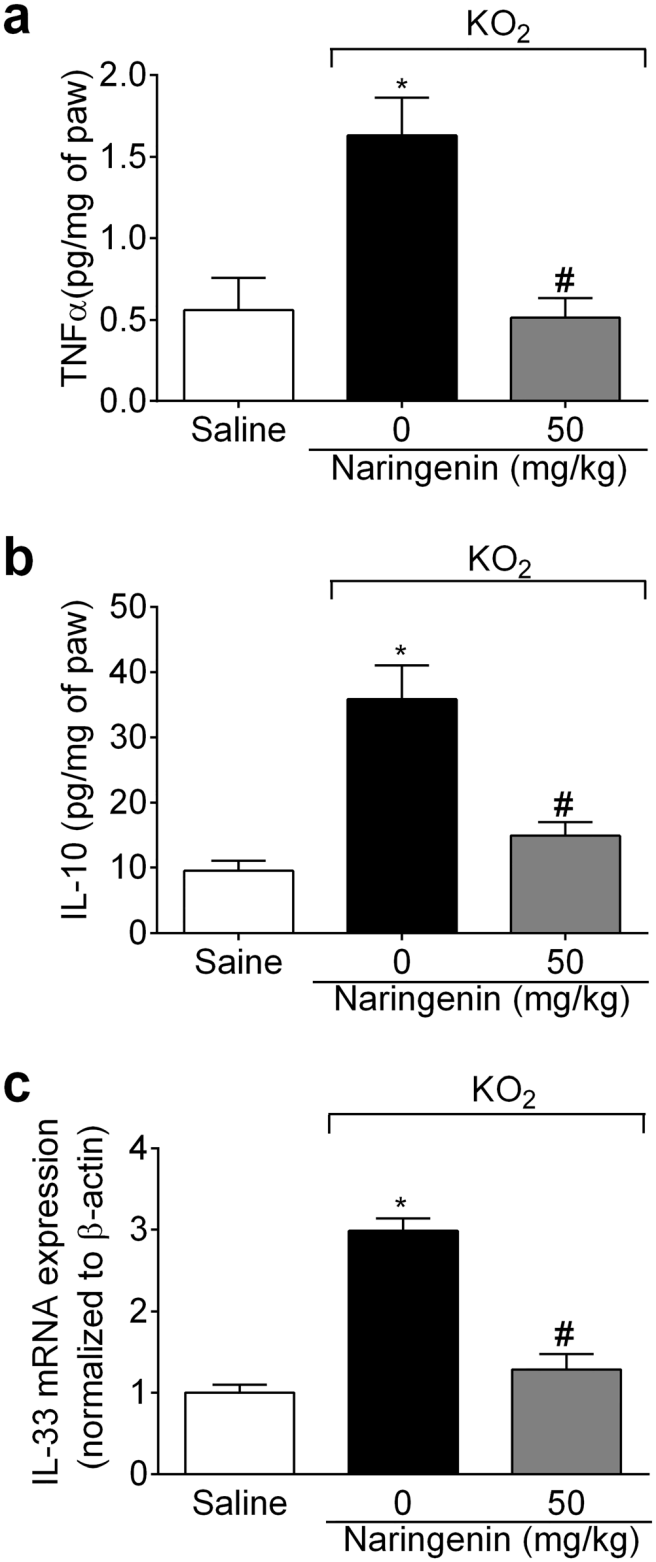
Naringenin inhibits KO_2_-induced cytokine production and mRNA expression. (a-c) Mice received naringenin (50 mg/kg, po) treatment 1 h before ipl injection of 30 μg of KO_2._ Paw skin samples were collected 3 h after ipl KO_2_ injection. Sample analyses were (a) TNFα, and (b) IL-10 production by ELISA, and (c) IL-33 mRNA expression by RT-qPCR. β-actin was a reference gene to normalize mRNA expression data. Results are mean ± SEM of 6 mice per group per experiment, and are representative of 2 independent experiments. *p< 0.05 vs. saline group, #*p*< 0.05 vs. KO_2_ group. One-way ANOVA followed Tukey’s post hoc.

The protocol of this section was the same as for Fig 4, with samples analyzed by ELISA and RT-qPCR. Naringenin treatment inhibited KO_2_-induced TNFα (Fig 5a) and IL-10 (Fig 5b) production, and IL-33 mRNA expression (Fig 5c).

### Naringenin inhibits KO_2_-induced COX-2 mRNA expression

**Fig 6:**
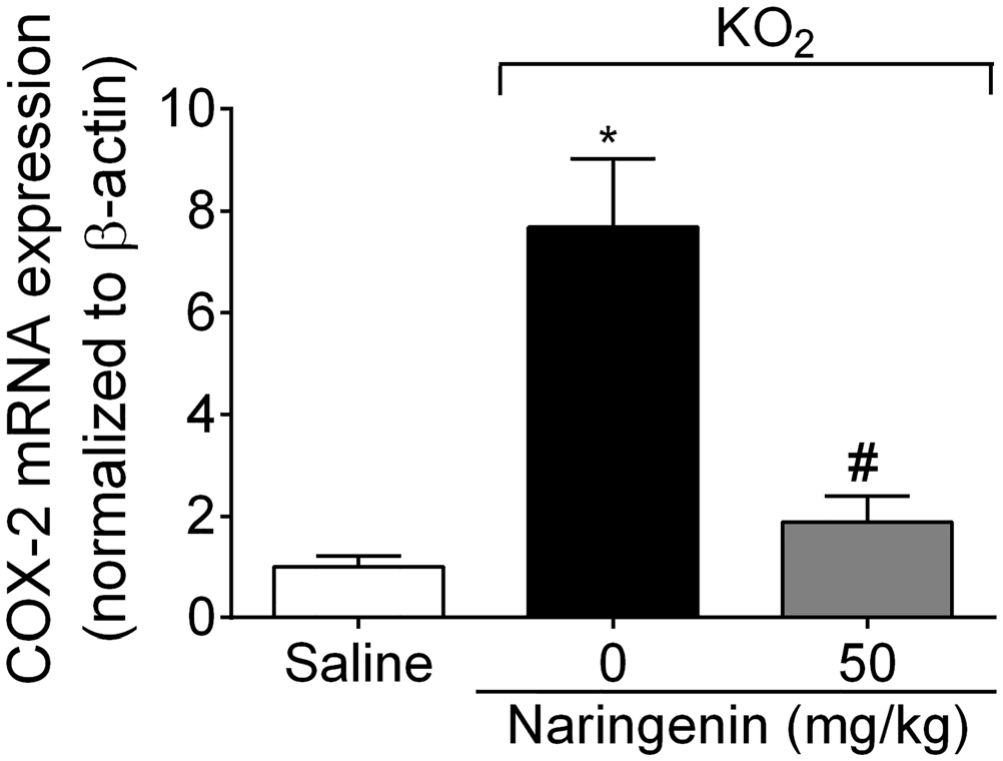
Naringenin inhibits KO_2_-induced COX-2 mRNA expression. Mice received naringenin (50 mg/kg, po) treatment 1 h before ipl injection of 30 μg KO_2._ Paw skin samples were collected 3 h after ipl KO_2_ injection and analyzed for COX-2 mRNA expression by RT-qPCR. β-actin was a reference gene to normalize mRNA expression data. Results are mean ± SEM of 6 mice per group per experiment, and are representative of 2 independent experiments. *p< 0.05 vs. saline group, #*p*< 0.05 vs. KO_2_ group. One-way ANOVA followed Tukey’s post hoc.

The protocol of this section was the same as for Fig 4, with samples analyzed by RT-qPCR. Naringenin treatment inhibited KO_2_-induced COX-2 mRNA expression (Fig 6).

### Naringenin inhibits KO_2_-induced preproET-1 mRNA expression

**Fig 7:**
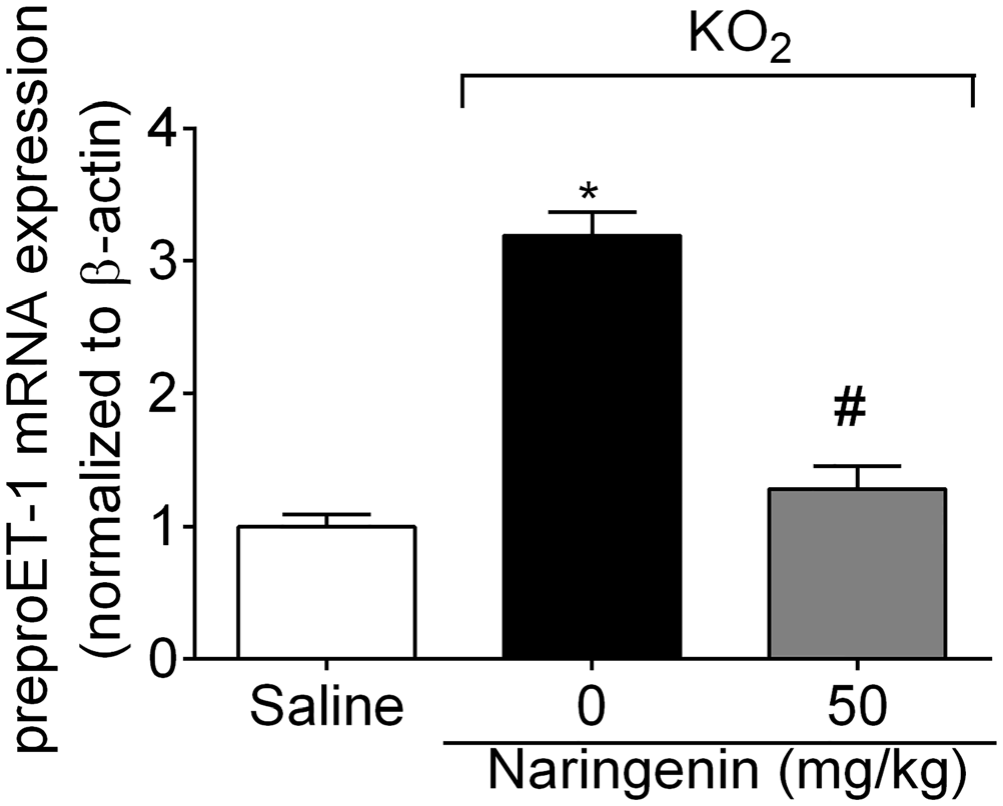
Naringenin inhibits KO_2_-induced preproET-1 mRNA expression. Mice received naringenin (50 mg/kg, po) treatment 1 h before ipl injection of 30 μg of KO_2._ Paw skin samples were collected 3 h after ipl KO_2_ injection for preproET-1 mRNA expression analysis by RT-qPCR. β-actin was a reference gene to normalize mRNA expression data. Results are mean ± SEM of 6 mice per group per experiment, and are representative of 2 independent experiments. *p< 0.05 vs. saline group, #*p*< 0.05 vs. KO_2_ group. One-way ANOVA followed Tukey’s post hoc.

The protocol of this section was the same as for Fig 4, and samples analyzed by RT-qPCR. Naringenin treatment inhibited KO_2_-induced preproET-1 mRNA expression (Fig 7).

### Naringenin increases Nrf2/HO-1 mRNA expression

**Fig 8:**
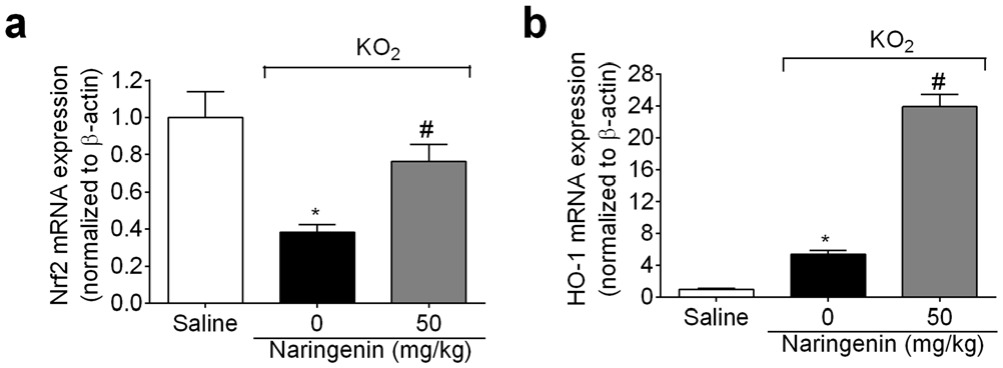
Naringenin increases Nrf2 and HO-1 mRNA expression. (a-b) Mice received naringenin (50 mg/kg, po) treatment 1 h before ipl injection of 30 μg KO_2._ Paw skin samples were collected 3 h after ipl KO_2_ injection for (a) Nrf2, and (b) HO-1 mRNA expression analysis by RT-qPCR. β-actin was a reference gene to normalize mRNA expression data. Results are mean ± SEM of 6 mice per group per experiment, and are representative of 2 independent experiments. *p< 0.05 vs. saline group, #*p*< 0.05 vs. KO_2_ group. One-way ANOVA followed Tukey’s post hoc.

The protocol of this section was the same as for Fig 4, with samples analyzed by RT-qPCR. Naringenin inhibited the KO_2_-induced decrease in Nrf2 mRNA expression (Fig 8a), whilst enhancing KO_2_-induced HO-1 mRNA expression (Fig 8b).

## Discussion

Pain is a multisensory experience, which occurs in response to stimulation of the channels/receptors that depolarizes nociceptor terminals, thereby generating an action potential, which activates synaptic transmission in the dorsal horn of spinal cord. Such processes induce a characteristic set of responses, including hyperalgesia (an increased response to a stimulus that normally provoke pain) and allodynia (pain due to a stimulus that does not normally provoke pain) [1].

Free radicals in biological systems are natural products during interactions between cells, as well as between tissues and organs. However, an imbalance between endogenous oxidants and antioxidants can alter cell homeostasis. Oxidants target proteins, thereby inducing cell damage and contributing to cell death [35]. Free radicals also cause inflammation [36]. Consequently, treatment with antioxidants is a promising approach to control a wide array of pathophysiological events. KO_2_ is a O_2_^−^ donor, and its injection resulting in nociceptive responses, such as overt pain-like behavior, as well as mechanical and thermal hyperalgesia, which can be inhibited by analgesics [6]. Data indicates that O_2_^−^-induced pain depends on both direct superoxide anion effects, and indirect effects, partly *via* the production of peroxynitrite [37]. O_2_^−^ injection triggers inflammatory pain by mechanisms involving cytokine, COX-2 and ET-1 synthesis [6,38,39]. Oxidative stress is involved in the pain of varied inflammatory diseases such as rheumatoid arthritis [40,41], gout [42], delayed onset muscle soreness [43] and diabetes [44]. Therefore, KO_2_-induced inflammatory pain is a useful model to study the action of analgesics that target oxidative stress-dependent events [12].

The therapeutic properties of naringenin include antinociception [15–17], as well as antioxidant [22,23,44–48] and anti-inflammatory activities [18,19,21,22]. However, the analgesic effect and mechanisms of action of naringenin in a model o oxidative stress-triggered inflammatory pain remained to be determined. Herein, we provide the first evidence that naringenin inhibits KO_2_-induced overt-pain like behavior, MPO activity, mechanical hyperalgesia and thermal hyperalgesia via reduction of cytokine production and oxidative stress.

The activation of the NO−cGMP−PKG−K_ATP_ channel signaling pathway leads to nociceptor hyperpolarization, thereby reducing nociceptor neuronal transmission [25–28]. The present results show that naringenin inhibits the KO_2_-induced mechanical and thermal hyperalgesia, at least in part, by activating the NO−cGMP−PKG−K_ATP_ channel signaling pathway. Free radical damage to membrane components leads to cellular dysfunction [49], which, by exposing neoepitopes, will contribute to an autoimmune response, which is receiving increasing interest as to its role in longer-term nociception. GSH is an important redox cellular system, with efficacy partly driven by the direct interactions of sulfhydryl groups (-SH) with ROS, thereby promoting a direct detox reaction [50]. Naringenin was able to restore GSH levels, as well as inhibiting other indicants of oxidative stress, such as FRAP, TBARS, and NBT in the paw skin. The KO_2_ solution releases O_2_^−^ for up to 10 min [6], however, NBT reduction (O_2_^−^ production) occurred in paw skin samples collected 3 h after KO_2_ injection. Therefore, O_2_^−^ injection induces further O_2_^−^. This is corroborated by the results showing KO_2_-induced the gp91^phox^ mRNA expression, which was inhibited by naringenin. The gp91^phox^ subunit of NADPH oxidase participates in the electron transfer to oxygen, thereby generating O_2_^−^ [51]. O_2_^−^-induced pain also depends on hyperalgesic cytokines, with TNFR1 deficiency reducing O_2_^−^-induced pain and oxidative stress. TNFα-induced hyperalgesia depends on NADPH oxidase activation [38]. Not all cytokines are nociceptive, with IL-10 having anti-hyperalgesic effects [43]. IL-33 is a hyperalgesic cytokine that regulates the production of TNFα and IL-10 [52–55] Naringenin inhibited O_2_^−^-induced production of TNFα and IL-10, as well as IL-33 mRNA expression.

Cytokine-induced inflammatory hyperalgesia in turn, depends, at least in part, on COX-2-dependent production of prostanoids such as prostaglandin E_2_ and prostacyclin [1]. O_2_^−^ also induces COX-2 mRNA expression, with celecoxib (a selective COX-2 inhibitor) diminishing O_2_^−^-induced pain [6]. Naringenin inhibited O_2_^−^-induced COX-2 mRNA expression. There is also a close relationship between cytokines and ET-1. Endothelin receptor antagonists inhibit cytokine-induced hyperalgesia and cytokines induce preproET-1 mRNA expression and ET-1 production [56,57]. Bosentan, an endothelin receptor antagonist, inhibits O_2_^−^-induced hyperalgesia, and O_2_^−^ induces preproET-1 mRNA expression [39]. Naringenin inhibited O_2_^−^-induced prepro-ET-1 mRNA expression. Furthermore, activation of the ET receptors promotes oxidative stress and reduces the free radical scavenging ability [58]. Therefore, it is likely that ET-1 also contributes to the regulation of oxidative stress. In fact, bosentan inhibited O_2_^−^-induced oxidative stress [39].

Consistent with the naringenin inhibition of cytokine production as well as COX-2 and preproET-1 mRNA expression, naringenin inhibits the activation of the pro-inflammatory transcription factor, NFκB, in several models of inflammation. For instance, naringenin inhibits NFκB activation in dextran sulphate sodium-induced colitis [59], ethanol-induced liver injury [18], streptozotocin-induced diabetes in mice [60] and rats [61], and experimental stroke [23]. Furthermore, naringenin also inhibits NFκB DNA-binding activity in ovalbumin-induced asthma [62] as well as in *in vitro* studies [63,64]. As such, it is not unlikely that naringenin inhibits KO_2_-induced cytokines production, as well as COX-2 and preproET-1, by inhibiting NFκB induction and activity. Nrf2 is an important transcriptional regulator of the antioxidant response. In fact, Nrf2 activation is essential to the production of endogenous antioxidants such as GSH, thioredoxin system, HO-1, and NQO1 (NAD(P)H dehydrogenase, quinone 1) [65]. Keap-1 detects cellular environment changes such as oxidative stress, resulting in the activation of Nrf2, which readily translocates to the nucleus and upregulates downstream targets such as HO-1 [66]. Furthermore, the activation of the Nrf2/HO-1 pathway inhibits the production of inflammatory molecules such as TNFα [67–69], IL-6 [68,69] and IL-1β [67,68]. Nrf2 indirectly modulates NFκB activity [70], given that Nrf2 and NFκB compete for binding to the nuclear complex, coactivator p300/CBP (E1A binding protein p300/CREB-binding protein) [71]. Importantly, naringenin activates Nrf2 and is an agonist of the aryl hydrocarbon receptor, contributing to reducing the production of reactive oxygen species and inflammatory mediators, as well as modulating specific immune cell activity [47,72]. In the present study, naringenin inhibited the KO_2_-induced decrease in Nrf2 mRNA expression and increased HO-1 mRNA expression. This mechanism might account for the analgesic effect of naringenin in KO_2_-induced inflammation, by inhibiting pro-hyperalgesic cytokine production, and gp91^phox^ as well as inducing antioxidant molecules.

The data presented here suggest that naringenin inhibits O_2_^−^-induced inflammatory overt pain-like behavior, hyperalgesia, and neutrophil recruitment by activating the NO−cGMP−PKG−K_ATP_ channel signaling pathway, as well as inhibiting oxidative stress and cytokine production, coupled to decreasing the mRNA of gp91^phox^, IL-33, COX-2, and preproET-1, and increasing Nrf2 and HO-1 mRNA expression. These data show for the first time that naringenin inhibits inflammatory pain triggered by O_2_^−^ and its mechanisms. Therefore, suggesting that naringenin is a promising therapeutic approach as an analgesic, antioxidant and anti-inflammatory compound, requiring further investigation in nociception, as well as in the array of other medical conditions where these pathophysiological changes are also evident [73].
